# Metal- and Organ-Specific Response to Heavy Metal-Induced Stress Mediated by Antioxidant Enzymes’ Activities, Polyamines, and Plant Hormones Levels in *Populus deltoides*

**DOI:** 10.3390/plants11233246

**Published:** 2022-11-26

**Authors:** Marko Kebert, Saša Kostić, Vanja Vuksanović, Anđelina Gavranović Markić, Biljana Kiprovski, Martina Zorić, Saša Orlović

**Affiliations:** 1 Institute of Lowland Forestry and Environment, University of Novi Sad, Antona Čehova 13d, 21000 Novi Sad, Serbia; 2 Faculty of Agriculture, University of Novi Sad, Trg Dositeja Obradovića 8, 21000 Novi Sad, Serbia; 3 Division for Genetics, Forest Tree Breeding and Seed Science, Croatian Forest Research Institute, Cvjetno Naselje 41, HR-10450 Jastrebarsko, Croatia; 4 Institute of Field and Vegetable Crops, National Institute of the Republic of Serbia, Maksima Gorkog 30, 21000 Novi Sad, Serbia

**Keywords:** cadmium, nickel, phytoremediation, plant hormones, polyamines, poplar, *Populus deltoides*

## Abstract

Besides anthropogenic factors, climate change causes altered precipitation patterns that indirectly affect the increase of heavy metals in soils due to hydrological effects and enhanced leaching (i.e., Cd and Ni), especially in the vicinity of mines and smelters. Phytoextraction is a well-known, powerful “green” technique for environmental clean-up that uses plants to extract, sequester, and/or detoxify heavy metals, and it makes significant contributions to the removal of persistent inorganic pollutants from soils. Poplar species, due to their growth features, high transpiration rate, large biomass, and feasible reproduction represent great candidates for phytoextraction technology. However, the consequences of concomitant oxidative stress upon plant metabolism and the mechanism of the poplar’s tolerance to heavy metal-induced stress are still not completely understood. In this study, cuttings of poplar species (*Populus deltoides* W. Bartram ex Marshall) were separately exposed to two heavy metals (Cd^2+^ and Ni^2+^) that were triple the maximum allowed amount (MAA) (according to national legislation). The aim of the study was to estimate the effects of heavy metals on: (I) the accumulation of free and conjugated polyamines, (II) plant hormones (including abscisic acid-ABA and indole-3-acetic acid-IAA), and (III) the activities of different antioxidant enzymes at root and leaf levels. By using the selected ion monitoring (SIM) mode of gas chromatography with mass spectrometry (GC/MS) coupled with the isotopically labeled technique, amounts of ABA and IAA were quantified, while polyamine amounts were determined by using high-performance liquid chromatography (HPLC) with fluorometric detection after derivatization. The results showed that *P. deltoides* responded to elevated concentrations of heavy metals in soils by exhibiting metal- and organ-specific tolerance. Knowledge about tolerance mechanisms is of great importance for the development of phytoremediation technology and afforestation programs for polluted soils.

## 1. Introduction

Two major global environmental problems—future climate change and heavy metal (HM) pollution—are cross-linked and co-dependent. Some climate scenarios predict altered precipitation patterns with an increasing trend of precipitation of up to 30% before 2030, which may result in increased heavy metal mobility and HM leaching into groundwater (depending on HM solubility) [1,2]. This is especially threatening due to the non-biodegradable, hazardous, and persistent nature of heavy metals that lead to their accumulation in the soil above allowed amounts and at toxic levels [3]. Although some heavy metals are essential for plants and humans in low amounts, their cumulative effects and biomagnification phenomenon (or the significant increase of metal content through the food chain as trophic levels rise) make them dangerous to animal and human health as they bioaccumulate in higher amounts [4,5,6].

Cadmium (Cd^2+^), in particular, accumulates in the human body (primarily in the kidneys), and it has a negative impact on many organs due to its high toxicity, causing pulmonary emphysema, renal tubular damage, and kidney stones [7]. Whereas nickel (Ni^2+^) has the potential to cause severe allergies, lung fibrosis, and even lung and nasal cancer by causing epigenetic alternation [8]. 

Although many HM in soils slithogenic origins, with some HM released during pedogenesis, two of the most notorious heavy metals, Cd and Ni, primarily enter the soil through anthropogenic activities, such as wastewater irrigation, zinc mining, automobile exhaust smoke, fossil fuel combustion, electroplating, industrial waste, pigments metal alloys, industrial or municipal waste, or excessive application of HM containing pesticides or synthetic phosphate fertilizers [5,9,10]. In addition, the recent overconsumption of electronic products has resulted in the increasing trend of electronic waste as a significant source of HM contamination and soil overload [11,12,13]. High amounts of toxic HM pollute the environment due to crude and unscientific e-waste recycling procedures (such as mechanical separation, hydrometallurgical, pyrometallurgy, etc.), particularly in the case of Ni-Cd batteries and their carcinogenic electrolytic waste, which make significant contributions to the leaching of toxic HMs in soils [14,15]. Therefore, there is an urgent and critical need for the development of new technologies for HMs clean-up from the environment since old traditional methods, such as excavation, heat treatment, electroremediation, chemical precipitation, metal leaching, and soil washing or replacement, are all rather expensive and invasive [16,17]. 

Phytoremediation refers to an environmentally friendly, aesthetically pleasing, and low-cost technology that uses plants (as well as their associated microbes) as solar-powered pumps to uptake, sequester, and/or detoxify organic or inorganic pollutants (including HMs, organic contaminants, radionuclides, antibiotics, pesticides, and even explosives such as trinitrotoluene, etc.) from various mediums (including soil, water, and air) and translocate them into harvestable parts [18,19,20]. Specifically, the process of soil restoration of HMs by plants is called phytoextraction [21,22], and it is the best-known technique of phytoremediation besides phytostabilization, phytodegradation, rhizofiltration, and phytovolatilization [16]. Although phytoremediation has been known for decades, it is still an emerging technique since upcoming climate change will impose new demands and necessitate new adjustments and improvements in terms of sustainability [18]. Crispr-Cas9, a new generation of plant genome editing technologies, provides tools for the rapid improvement of phytoremediation technology by designing genome engineered metallicolous plants with improved heat and drought tolerance, specifically for the purpose of sustainable phytoremediation [23]. Although many herbaceous plants (e.g., *Dysphania botrys*, *Lotus corniculatus*, *Lotus hispidus*, *Plantago lanceolata*, *Trifolium repens*, and *Medicago lupulina*) exhibit metallophyte behavior and are efficient regarding metal accumulation and translocation [24], wooden plant species are more appropriate for phytoremediation due to their large biomass production [25]. Poplars and willows are particularly suitable for phytoextraction because they are fast-growing species with high transpiration indices, easy propagation technology, and a deep rooting system, as well as a high ability to accumulate and translocate essential and non-essential HMs into aerial parts [26,27,28,29]. 

Recently, the entire genome of *Populus trichoderma* has been sequenced, making poplars especially appealing candidates for genome editing toward HM stress tolerance and further investigation of its potential to act as an efficient phytoremediator [23,30]. In this study, we used eastern cottonwood (*Populus deltoides* W. Bartram ex Marshall) clone PE19/66 to investigate Ni and Cd effects on *P. deltoides* biochemical properties since it has been shown to be particularly tolerant to copper by accumulating high amounts of proline (PRO) and abscisic acid (ABA) in its leaves and roots and high amounts of polyamines in its roots during HM induced stress [31].

Understanding the underlying mechanisms of HM stress tolerance is critical for the further development of phytoremediation techniques, the selection of the most suitable clones, and potential gene targeting and editing. Through evolution, plants evolved their entire machinery to combat HM pollution, and they created/employed strategies and mechanisms to sequester and detoxify HM in order to reduce their toxicity [32,33,34]. Cd has no known biological functions in higher plants and does not participate in redox reactions, but it does contribute to oxidative damage, protein carbonylation, and lipid peroxidation, and it is extremely toxic to plants due to its high affinity for protein sulfhydryl groups, thus causing the inhibition of many enzymes [35,36]. In contrast, nickel is a structural component of many enzymes, including glyoxylases, peptide-deformylases, methyl-CoM reductases, and several types of superoxide-dismutases and hydrogenases [37]. Excess Ni causes Ni-toxicity, and symptoms (such as leaf chlorosis, plant root growth inhibition, and decreased photosynthesis and respiration) may appear, and Ni also disrupts mineral nutrition, water relations, and sugar transport [38]. Heavy metals, such as Cd and Ni, are uptaken by roots via the apoplastic (passive diffusion) or symplastic (active transport by root plasma membrane transporters for essential elements with low selectivity) pathways, where they form complexes with different chelating agents and are then immobilized in cell walls and vacuoles where detoxification occurs, either by conjugating with glutathione (GSH) or cysteine-rich peptides, such as phytochelatins (PC) or metallothioneins (MS) [39,40]. Heavy metal ions and essential metal ions have a similar radius and charge; therefore, heavy metals can impair the uptake and transport of essential metals like calcium and magnesium [41]. 

Since heavy metals cause oxidative stress in plants by increasing the production of reactive oxygen species (ROS), the activation of ROS scavenging enzymes, such as superoxide dismutase (SOD), catalase (CAT), ascorbate peroxidase (Apx), glutathione reductase (GR), thioredoxin, and the peroxy-redoxin family of proteins, is one of the first lines of defense [31,42,43]. In addition to enzymatic, antioxidant defense also includes non-enzymatic ROS scavengers, such as ascorbate and glutathione, carotenoids, tocopherols, quinones, lipoic acid, phenolic compounds, polyamines, etc. [44,45].

Polyamines have a variety of regulatory roles in plant cells due to their antioxidant and polycationic nature, and as important abiotic stress markers, they modulate plant tolerance to HM through the direct scavenging of ROS or the activation of antioxidant machinery, or they act as signal molecules to activate ABA or H_2_O_2_ stress-responsive pathways [46,47,48]. Their protective role in HM stress has already been proposed, and increasing patterns of both free and conjugated polyamines (such as putrescine, spermidine, and spermine) have been reported as a poplars response to copper and zinc, but to the best of the authors’ knowledge, they have not been examined in response to nickel and cadmium [31,49,50]. 

Although it is well known that abscisic acid (ABA), which is an important plant stress hormone, and indole-3-acetic acid (IAA), which is a developmental hormone, are involved in the perception and signaling of excess HMs by roots, these hormones also affect plant growth and regulate the antioxidant defense system in the presence of HMs [31,51,52]. In particular, their exogenous application prevents the negative effects of excess HM on plant growth and overall fitness [53,54]. Still, little is known about how Ni and Cd affect endogenous plant hormone levels and distribution in *P. deltoides*.

Therefore, the main aim of the study was to investigate how excess amounts of Cd and Ni in soil affect *P. deltoides* responses at root and leaf levels, regarding:

✓ The metal content (calcium and magnesium), translocation (TF), and bioconcentration factors (BCF) in *P. deltoides* clone Pe19/66;
✓ The activities of different ROS scavenging enzymes, such as guaiacol peroxidase, glutathione reductase, and superoxide dismutase;
✓ Total antioxidant and reducing activities (estimated by biochemical assays DPPH and FRAP, respectively) and radical scavenger capacity (against NO and OH radicals), as well as total polyphenol compounds (TPC) accumulation; and
✓ Endogenous hormone levels (ABA and IAA), as well as plant hormone, regulators-polyamines content (putrescine, spermine, and spermidine) that is both free and conjugated.

## 2. Results

### 2.1. Metal and Non-Metal Contents, Translocation (TF), and Bioconcentration Factors (BCF)

The results regarding the contents of root and shoot uptake of metal content, calcium and magnesium levels, and bioconcentration and translocation in *P. deltoides* clone 19/66 leaves and roots are presented in Table 1. 

### 2.2. The Effects of Cd and Ni on Antioxidant Enzymes Activities in Poplar Leaves and Roots

**Figure 1:**
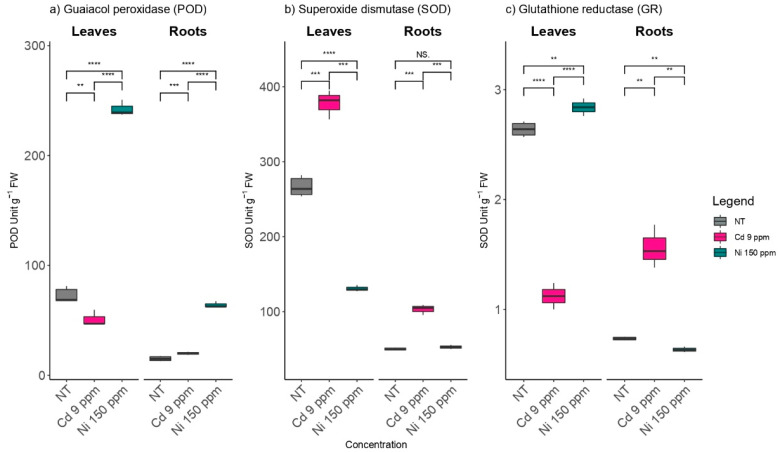
The effects of elevated Cd and Ni soil contents on (**a**) guaiacol peroxidase (POD); (**b**) superoxide dismutase (SOD), and (**c**) glutathione reductase (GR) activities at root and leaf levels using NT-non treated soil. Cd 9 ppm-soil was supplemented to 9 mg Cd kg^−1^ of soil DW and Ni 150 ppm-soil was supplemented to 150 mg Ni kg^−1^ of soil DW. Significance levels: (NS) non-significant versus significant (**) < 0.01; (***) < 0.001, and (****) < 0.0001.

Activities of POD, SOD, and GR were significantly higher in the leaves than in the roots of the tested poplar plants (Figure 1). POD activity in the poplar leaves was 30% lower in the Cd (9 ppm) treatment compared with the unpolluted control, while GR activity was 57% lower in the *P. deltoides* plants under Cd treatment compared with the untreated controls (Figure 1a,c). The highest level of SOD (377.7 SOD g^−1^ FW) activity was observed in the leaves of *P. deltoides* treated with Cd (9 ppm) (Figure 1b). *P. deltoides* cuttings treated with Ni (150 ppm) had significantly higher foliar POD activity (242.4 POD g^−1^ FW). There was no statistically significant difference in SOD and GR activities in roots treated with Ni (150 ppm) when compared to those from the untreated control, contrary to the activity of POD, which was significantly higher in Ni-treated roots (Figure 1).

### 2.3. The Effects of Cd and Ni on the Antioxidant Capacity of Poplar Leaves and Roots

**Figure 2:**
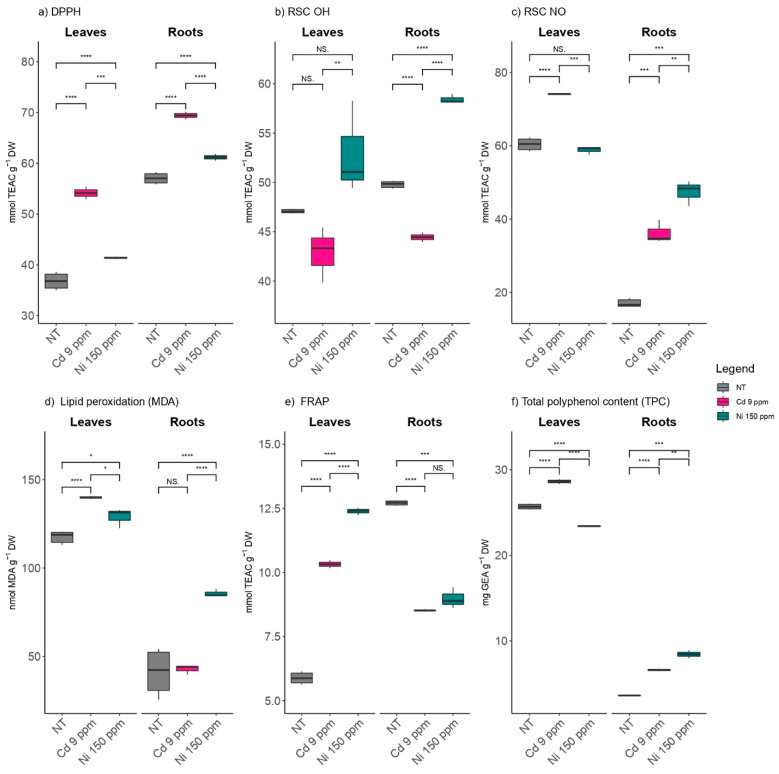
The effects of elevated Cd and Ni soil contents on radical scavenger activities against (**a**) DPPH, (**b**) OH, and (**c**) NO radicals, as well as (**d**) lipid peroxidation, (**e**) ferric reducing antioxidant power (FRAP), and (**f**) total polyphenol content (TPC) at root and leaf levels using NT-non treated soil. Cd 9 ppm-soil was supplemented to 9 mg Cd kg^−1^ of soil DW and Ni 150 ppm-soil was supplemented to 150 mg Ni kg^−1^ of soil dry weight. Significance levels: (NS) non-significant versus significant (*) < 0.05; (**) < 0.01; (***) < 0.001; and (****) < 0.0001.

The highest scavenging capacity against DPPH radicals (64.9%) was measured in the extracts of poplar roots grown in the soil treated with Cd (9 ppm). In general, a significantly higher scavenging capacity against DPPH radicals was detected in the leaf extracts of *P. deltoides* from all the examined treatments compared to the control, both in the extracts of the roots and in the leaves (Figure 2a). Scavenger capacity against NO radical indicates that the ability to neutralize NO radicals increases under the influence of Ni and Cd ions since the values for NO scavenger capacity ranged from 17% in the extracts of the roots from the control up to 74.1% in the extracts of the leaves from the plants grown using the treatment with Cd (9 ppm) (Figure 2b). Cd treatment at a concentration of 9 ppm did not affect the ability of *P. deltoides* leaves and root extracts to neutralize OH radicals, yet plants treated with 150 ppm Ni had a significantly higher capacity compared to those from the control (Figure 2c). The ability of leaves extracts to neutralize NO and OH radicals were more affected by the Ni treatment (150 ppm), while the ability to neutralize DPPH radicals was more affected by treatment with Cd (9 ppm) (Figure 2a–c). 

After the application of Ni and Cd in amounts that were three times higher than the MAA, there was an increase in LP intensity in both the roots and the leaves (Figure 2d). The highest LP intensity was observed in the poplar leaves grown using the treatment with Cd (9 ppm) (139.9 nmol MDA g^−1^ DW). In general, MDA accumulation was higher in the leaves than in the roots, but the difference in MDA content was higher in the roots when control and treatments were compared. Also, Ni treatment had a greater effect on MDA accumulation than Cd treatment.

The reducing capacity of the *P. deltoides* leaves extracts increased by 100% in Ni (150 ppm) and Cd (9 ppm) treatments compared with the control, while the reducing capacity of the roots extracts decreased by about 30% compared with the control (Figure 2e).

The content of polyphenol compounds (TPC) in poplar leaves ranged from 23.4 mg GAE g^−1^ DW (Ni 150 ppm) to 28.6 mg GAE g^−1^ DW (Cd 9 ppm). The highest TPC content was measured in the leaves of the plants from the Cd treatment (9 ppm), and the lowest was detected in the roots of the plants grown using the control media. Significantly higher values of TPC under the influence of Ni ions (150 ppm) were measured in poplar roots compared with the control (Figure 2f ).The increase in the content of phenolic compounds was more pronounced in the roots than in the leaves. 

### 2.4. The Effects of Cd and Ni on Plant Hormones and Hormone Regulators Content

**Figure 3:**
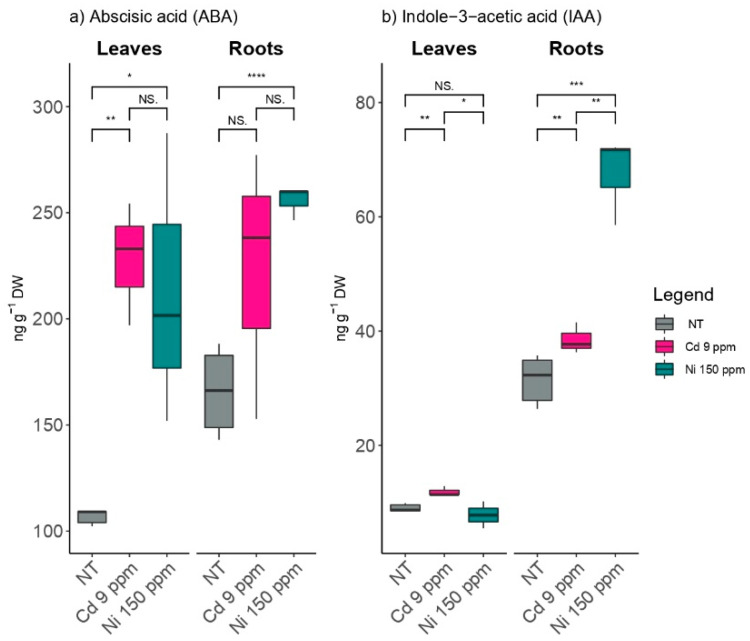
The effects of elevated Cd and Ni soil contents on amounts of plant hormones, (**a**) abscisic acid (ABA), and (**b**) indole-3-acetic acid (IAA) at root and leaf levels using NT-non treated soil. Cd 9 ppm-soil was supplemented to 9 mg Cd kg^−1^ of soil DW and Ni 150 ppm-soil was supplemented to 150 mg Ni kg^−1^ of soil DW. Significance levels: (NS) non-significant versus significant (*) < 0.05; (**) < 0.01; (***) < 0.001; and (****) < 0.0001.

The content of abscisic acid increased under the influence of both applied metals, (Ni and Cd increased by 100% and 114%, respectively), whereas the levels of abscisic acid detected in the roots did not differ significantly under the metal treatments (Figure 3a). The constitutive levels of abscisic acid in the roots of the untreated plants were higher than in the leaves.

Similarly, the roots had a significantly higher content of indole-3-acetic acid than the leaves (Figure 3b). The nickel treatment significantly increased IAA levels in the roots, but there were no significant changes in foliar IAA under either treatment.

**Figure 4:**
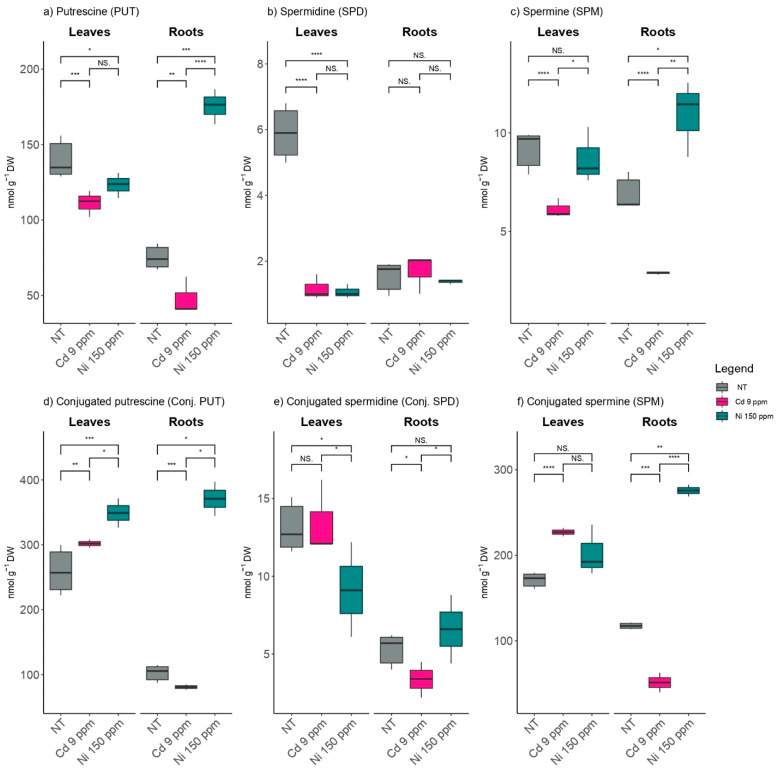
The effects of elevated Cd and Ni soil contents on amounts of accumulated free polyamines ((**a**) putrescine-PUT, (**b**) spermidine-SPD, and (**c**) spermine-SPM), as well as conjugated polyamines ((**d**) conjugated putrescine-conj. PUT, (**e**) conjugated spermidine-conj. SPD, and (**f**) conjugated spermine-conj. SPM) at root and leaf levels using NT-non treated soil. Cd 9 ppm-soil was supplemented to 9 mg Cd kg^−1^ of soil dry weight and Ni 150 ppm-soil was supplemented to 150 mg Ni kg^−1^ of soil DW. Significance levels: (NS) non-significant versus significant (*) < 0.05; (**) < 0.01; (***) < 0.001; and (****) < 0.0001.

The *P. deltoides* clone Pe19/66 showed a wide range of polyamine responses to different heavy metals (Cd and Ni) in terms of polyamines contents (including putrescine-PUT, spermidine-SMD, and spermine-SPM) in their free and conjugated forms and in organ (root and leaf) defined/related responses (Figure 4). Putrescine was the most abundant polyamine in both the free and conjugated fractions, with SPM being the least abundant in the analyzed *P. deltoides* clone. *P. deltoides* exposed to increased Ni concentrations resulted in a significant increase in PUT and SPM at the root level, but no significant changes in SPD levels occurred. At the leaf level, a different pattern was observed. Ni induced a significant reduction in SPD when compared to non-treated controls, whereas foliar PUT and SPM did not change under the influence of Ni ions. When exposed to excess Ni, all conjugated polyamines increased significantly at the root level, while at the leaf level, conjugated SPD decreased and foliar conjugated PUT and SPM increased slightly compared to untreated controls. Under Cd treatment, all free polyamines exhibited significant declining trends in both inspected organs compared to non-treated controls, with the exception of SPD at the root level, which remained unchanged compared to non-treated controls. Under Cd effects, all conjugated polyamines decreased at the root level compared to untreated *P. deltoides*, whereas foliar conjugated PUT and SPM increased compared to untreated controls, and conjugated SPD remained unchanged.

### 2.5. The Principal Component and Correlation Analysis 

**Figure 5:**
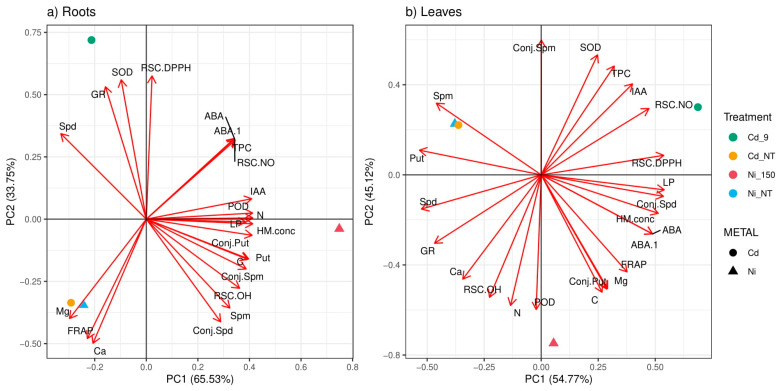
Principal Component Analyses (PCA) with treatment and heavy metal treatment as a dependent variable separately using root (**a**) and leaf (**b**) samples. Treatments include: Cd 9 (*P. deltoides* grown in soil supplemented with 9 ppm Cd); Cd_NT (poplar cuttings grown in non-treated soil); Ni 150 (poplar cuttings grown in soil supplemented with 150 ppm of Ni); and Cd_NT (poplar cuttings grown in Cd non-treated soil). The following abbreviations examined parameters. TPC: total phenolic content; FRAP: ferric reducing antioxidant power; LP: lipid peroxidation; SPD: spermidine; SPM: spermine; PUT: putrescine; SOD: superoxide dismutase; POD: guaiacol peroxidase; GR: glutathione reductase; ABA: abscisic acid; IAA: indole-3-acetic acid; and DPPH: 2,2-Diphenyl-1-picrylhydrazyl radical.

The Principal Component Analysis (PCA) of analyzed parameters (metabolites, enzymatic activities, radical scavenger activities, and metal content) separately for root (Figure 5a) and leaves (Figure 5b) samples showed differences in organ-specific manner to heavy metal stressors. In both analyzed organs, the first two principal components (PC) described mostly all sample variation (root: 99.28% and leaf: 99.89%). In the root samples (Figure 5a), parameters associated with PC1 (LP < N < POD < HM conc. < IAA < Put < Conj. Put) defined Ni induced stress, while parameters defined by PC2 (RSD DPPH < SOD < GR < Spd) were defined by Cd induced stress responses. In contrast, analyzed parameters in leaf samples showed that parameters associated with PC2 (POD < N < C < Conj. Put < RSC OH < Ca < Mg) defined Ni induced stress. Opposite to the parameter distribution in Figure 5a for root samples, Cd induced stressors were defined by both PCs, mostly with parameters which defined enzyme activity and hormonal status, particularly with parameters RSC NO < IAA < TPC < SOD < RSD DPPH. Firstly, non-treated control samples were closely distributed on both PCAs, while treated samples were obviously removed from each other as well as from the control samples. Different PCAs and correlation matrix patterns that define different relations among analyzed parameters indicate organ specific responses to elevated soil HM amounts.

Heavy metal root content positively correlated with almost all the measured parameters. We noted the strongest correlations between heavy metal content from roots with N and C contents, TPC and LP, enzymatic activities, hormonal status (ABA and IAA), and free polyamine content (PUT and SPM), as well as all conjugated forms of polyamines, and with parameters of antioxidant defense system (RSC NO and RSC OH). Root HM contents had a strong, negative correlation with SPD. In contrast to the roots, heavy metal contents in the leaves did not exhibit a uniform response. Although a majority of parameters showed a positive correlation, all inspected polyamines (PUT, SPD, and SPD) expressed negative correlations to HMs content. 

**Figure 6:**
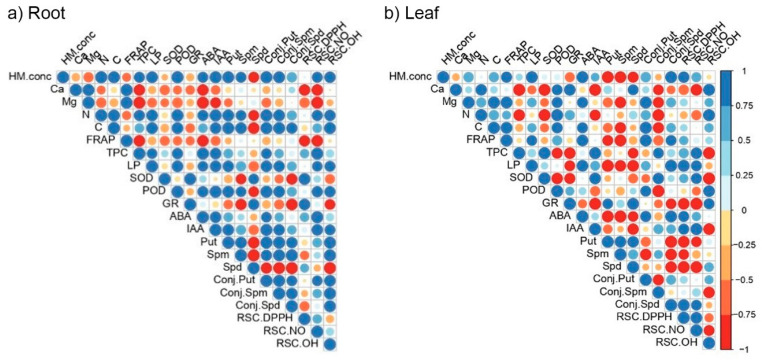
Pearson’s correlation matrix of all analyzed parameters for roots (**a**) and leaves (**b**) exposed to increased levels of Ni and Cd. The following abbreviations examined the parameters. TPC: total phenolic content; FRAP: ferric reducing antioxidant power; LP: lipid peroxidation; SPD: spermidine; SPM: spermine; PUT: putrescine; SOD: superoxide dismutase; POD: guaiacol peroxidase; GR: glutathione reductase; ABA: abscisic acid; IAA: indole-3-acetic acid; and DPPH: 2,2-Diphenyl-1-picrylhydrazyl radical.

Plant hormones (ABA and IAA) measured in both organs (leaves and roots) exhibited similar relation patterns as other analyzed parameters. We noted that ABA from root tissue was strongly and positively correlated with parameters of enzymatic activity and parameters of antioxidant defense system, such as TPC, LP, POD, and RSC NO, as well as with heavy metal content. In contrast, root levels of ABA were negatively correlated with FRAP values, Ca, and Mg contents, while the correlation with polyamines was missing. Root amounts of IAA showed similar patterns, such as ABA, with all parameters measured in the root tissue. Foliar plant hormones (ABA and IAA) obtained strong correlations with HM content applied using the treatments as well as with Mg and C content, FRAP values, LP activity, and conjugated forms of polyamines. In contrast, foliar ABA exhibited a strong negative correlation with free polyamines (PUT, SPM, and SPD) (Figure 6a).

Within polyamines detected from the root tissue, PUT and SPM showed similar correlation patterns as other examined parameters, contrary to SPD patterns. Likewise, PUT and SPM had strong mutual correlation at the root level. To expand, PUT and SPD in roots were positively correlated with applied amounts of heavy metals, C and N contents, and their conjugated forms, as well as with RSC OH and POD, while negative correlations were noted among PUT and SPM with SOD, Gr, and SPD. We observed opposite patterns of correlation among PUT, SPD and SPM at the leaf level compared with the root tissues, which contributed to the hypothesis of organ-specific responses to increased heavy metal content. At the leaf level, polyamines established numerous negative correlations, like those with HM contents, LP, ABA, RSC DPPH, and RSC NO, as well as conjugated forms of SPD (Figure 6b). Conjugated forms of polyamines exhibited different correlation patterns compared to their free forms extracted from leaves.

Parameters of the antioxidant defense system in roots (RSC NO and RSC OH) had similar relations as other analyzed parameters. The stronger positive correlations were noted with Ca, N, and C contents compared with LP, POD, IAA, and polyamines (PUT and SPM). This trend was opposite to the established correlations with parameters such as GR and SPD, which exposed negative correlation patterns only with RSC OH in the roots. Ambiguous and inconsistent patterns of antioxidant defense system correlations were detected at the leaf level. The most positive relationship was observed between RSC OH and Ca, N, and POD, while RSC NO negatively correlated with TPC, SOD, and IAA in poplar leaves (Figure 6a).

## 3. Discussion

### 3.1. The Effects of Cd and Ni on the Antioxidant Defense System

Heavy metals, in addition to influencing enzyme expression, also alter enzyme catalytic function due to their strong binding affinity to sulfhydryl or other groups from the enzyme’s active center, resulting in lower enzyme activity or even complete inhibition [55]. Heavy metals can interfere with the function of many enzymes and even displace important metal ions from active sites, resulting in altered activities or the loss of activity [56]. Excessive heavy metal amounts in plants cause oxidative stress which stimulates activities or upregulates expression patterns of defense antioxidant enzymes, such as superoxide dismutase (SOD) and glutathione reductase (GR) [57,58]. The results provided in this study reveal that the change in Ni content in poplar root extract had no effect on the activity of SOD and GR, but the results also confirm that Ni ions have stimulating effects on POD activity. In contrast, Cd ions boost the activity of SOD, POD, and GR in poplar leaf extracts. It was reported previously that Cd influences enzyme activity in poplars, resulting in higher activities of ascorbate peroxidase, glutathione peroxidase, and catalase in roots [59]. Since Ni is not a redox-active element, it is not expected to have a direct impact on the generation of reactive oxygen species; however, this property allows Ni ions to indirectly interact with a large number of antioxidant enzymes, such as superoxide dismutase (SOD), catalase (CAT), glutathione peroxidase (GSH-Px), glutathione reductase (GR), guaiacol peroxidase (GPx), and ascorbate peroxidase (Apx) [60]. The activity of antioxidant enzymes varies depending on the time of exposure, the type of treatment, and the species and plant organs involved [61]. According to Gajewska and Sklodowska [62], SOD and CAT activities decreased significantly in wheat leaves after treatment with 100 mM Ni for three, six, and nine days, while glutathione peroxidase (GSH-Px), guiacol peroxidase (POD), and ascorbate peroxidase (Apx) activities increased. However, the same authors [63] reported that exposing peas (*Pisum sativum*) to nickel ions for 14 days (concentrations of 10, 100, and 200 mM) reduced SOD activity in both shoots and roots, which is in accordance with our findings for poplar clone PE19/66. 

The results of the experiment show an increase in total polyphenol content in response to higher Ni and Cd ion concentrations. Similarly, a metal induced increase of total phenolics was reported for other plant species, such as corn [64], cress (*Lepidium sativum*) [65], Scots Pine (*Pinus sylvestris*) [66], and wheat (*Triticum sp*.) [67]. Furthermore, the content of phenolic chemicals increases in poplar as a result of Cd exposure. A concentration of 200 μM caused a 47% increase in phenolic compounds in the roots of *Populus deltoides* and a 38–168% increase in the bark of *Populus ×euramericana*, *Populus nigra*, and *Populus popularis*, while in the leaves of *Populus nigra,* phenolic content was 67% higher compared to control plants [59]. Phenolic compounds increase plant tolerance to various abiotic stress factors, such as temperature fluctuations, the presence of heavy metals [68], and water deficit [69,70], in addition to their importance in allelopathic relationships and herbivore defense [71]. Furthermore, other publications revealed that the total polyphenolic content of many plants reduced during abiotic stresses [72,73,74,75]. The defensive role of phenolic compounds is attributed to the photoprotective, osmoregulatory, and mostly antioxidant properties of these compounds [76]. According to the study’s findings, higher phenolic compound contents in roots and leaves reveals a greater ability to remove ROS. Increased Ni and Cd ion concentrations (three times higher than the MAA) increase the antioxidant capacity of treated poplar clones. There is an increase in the ability to neutralize DPPH radicals under the influence of Ni ions at a concentration of 150 ppm. This is most likely due to the activation of antioxidant defense, which is manifested via the increased biosynthesis of secondary metabolites under stressed conditions caused by Ni ions. Kebert et al. [77] investigated oxidative stress in the leaves of poplar clones Pe19/66, B229 (*P. deltoides*), and Panonnia (*P. ×euramericana*) after field exposure to a mixture of heavy metals (Ni, Cd, and Pb), herbicides, diesel fuel, and combined treatment with diesel and heavy metals. The authors stated that heavy metal treated poplar leaves had higher antioxidant capacity than the control group and identified clone B229 as the most tolerant to the treatments used. 

In addition, Cd induced stress activated the *Brassica juncea* antioxidative defense system [78]. However, the findings of a study about the toxic effects of cadmium on *Brassica rapa* var. *turnip* discovered that Cd treated plants had lower antioxidant activity [79]. The findings of this study reveal that the ferric reducing antioxidant power (FRAP) of poplar root and leaves extracts is increased when it is exposed to high levels of Ni and Cd. Furthermore, Kebert et al. [77] demonstrated that there is an increase in poplar leaf reducing capacity (estimated by FRAP assay) due to the exposure of plants to stress caused by heavy metals, pesticides, and/or diesel in the soil. The results of the FRAP test on the antioxidant capacity in basil (*Ocimum basilicum*) leaves revealed that the antioxidant capacity increased with a treatment of Ni 500 pp and that antioxidant capacity decreased as Ni content increased [80]. 

The results obtained in this research indicate that the amounts of measured malondialdehyde (MDA) in poplar leaves and roots increases with an increase of the Ni and Cd ions concentration. Previously published research revealed that the process of lipid peroxidation is enhanced in higher plants under situations of oxidative stress produced by heavy metals. As a result of heavy metal exposure, the amounts of MDA, as an end product of lipid peroxidation, increased in peas [81], different genotypes of rye (*Secale cereale*) [82], sunflower (*Helianthus annuus*) [83], *Arabidopsis thaliana* [84], nodules of soybean (*Glycine max* L.) [85], spring barley (*Hordeum vulgare* L.) [86], and citrus (*Citrus aurantium* L.) [87], which is consistent with the results of this research. 

### 3.2. HM Induced Stress Affected Plant Developmental and Stress Hormones (IAA and ABA) 

Abscisic acid (ABA) is a multifunctional phytohormone that has been linked with tolerance to adverse environmental conditions, and its signaling pathway is a key regulator of abiotic stress response in plants, including heavy metal induced stress [31,88,89,90]. It has been proposed that ABA accumulation and the regulation of ABA biosynthetic gene expression contribute to heavy metal tolerance without affecting growth [91]. The beneficial effects of ABA are associated with its ability to cause stomata closure and to regulate hydraulic conductivity during drought stress or during significant osmotic changes, such as those caused by HM [92]. Furthermore, ABA is involved in the regulation of genes encoding biosynthetic enzymes of different osmoprotective compounds, such as proline and glycine betaine [93,94]. The accumulation of ABA and proline is crucial in the development of tolerance to Cd ions, indicating the networking of signaling pathways in conditions of abiotic stress caused by water deficit and an increased content of heavy metals [95]. Improved tolerance in HM-stressed plant species has also been linked to exogenous ABA application [54]. In contrast, the endogenous levels of plant hormones changes after poplar plants are exposed to a high amount of Cd and Ni in the soil and tissue accumulation of this metal. The significant increase of ABA levels at the root level found in this study could be associated with the important role of this hormone in stress perception and root–shoot signal transduction that enables the transfer of information about increased contents of heavy metals in the soil to the shoots [96]. Our findings about increasing ABA root content are consistent with those found in *Phaseolus vulgaris* under Ni induced stress [97]. When plants are exposed to an excess of Cd ions, they exhibit symptoms of general plant stress, such as decreased leaf elongation and growth and decreased cell size (ethylene response), and symptoms of water deficit, such as decreased stomatal conductance and transpiration, which represent a typical ABA response since it is well known that toxic trace metals impair plants’ water balance [98,99,100]. Our findings show that Cd treatment increases ABA levels by 117% which aligns with previous studies that confirmed Cd induced ABA and ethylene biosynthesis in roots [101,102,103,104,105]. 

Furthermore, recent findings in Mung bean (*Vigna radiata*) after exogenous application of ABA during Cd stress demonstrated that ABA plays a role in HM tolerance via the regulation of antioxidant machinery [98]. This finding is consistent with the high positive correlations among ABA, total phenolic content, and peroxidase activity at the root level found in this study. Transpiration, on the other hand, stimulates Cd ion transport to the shoots, and exogenous ABA application reduces this transport [106,107]. ABA-mediated osmotic stress and ABA-mediated signaling pathways induced by excess Cd ions resulted in an increased expression of metallothionein in peas, indicating the existence of signaling crosstalk between drought and Cd induced stress [108]. Depending on the applied concentation, Ni ions can stimulate and inhibit the activities of enzymes involved in the metabolism of plant hormones. Thus, under the influence of 50 μmol NiCl_2_, the activity of indole-3-acetic acid oxidase in *O. sativa* seedlings significantly increased, while at higher concentrations of Ni ions, the enzymatic activity of this enzyme significantly decreased [109]. In our study, slightly increased root levels of IAA under both elevated Ni and Cd ions are present, while there are no significant changes in IAA at the leaf level. In contrast to our findings, arsenic (As) decreased levels of three auxins, including IAA, NAA, and indole-3-butyric acid (IBA), occurred in *Brassica juncea* [110], whereas short-term cadmium exposure also reduced IAA levels in the root tips of barley *(Hordeum vulgare)* [111].

### 3.3. Polyamines Exhibited Metal and Organ Specific Responses to HM-Induced Stress 

Polyamines, as ubiquitous polycationic antioxidants, have been shown to be mediators of increased heavy metal tolerance in numerous plant species by mitigating the toxic effects of heavy metals in plants [112]. Their protective role is based on their high antioxidant and strong ROS scavenger capacity, and therefore their ability to regulate redox homeostasis during oxidative stress caused by heavy metals [113,114], and also on their ability to act as metal chelators [115]. During their catabolism, polyamines generate hydrogen peroxide, allowing them to modulate entire ROS signaling pathways [116], but they can also activate plant antioxidant defense machinery, specifically affecting the gene expression of ROS scavenging enzymes [117,118]. As positively charged compounds, they have a high affinity for binding to negatively charged biomolecules, such as DNA or lipid membranes, increasing their stability and inhibiting lipid peroxidation, while also having a high affinity for binding to ionic channels and regulating ion homeostasis and ion transport in plants [119,120]. Depending on their charge (Spm^4+^ > Spd^3+^ > Put^2+^), polyamines block fast-activating vacuolar cation channels which gives them the ability to modulate salt stress tolerance in plants and heavy metal induced stress through modulation of metal transporters [121]. Increased tolerance to heavy metal induced stress has been linked to plants’ ability to increase endogenous levels of specific polyamines [31,122] or to the exogenous application of Pas during exposure to elevated heavy metal amounts in soil [123]. In our study, after long-term exposure to high Ni and Cd soil levels, polyamines exhibit organ- and metal-specific responses, with mostly decreasing patterns of free polyamines with increasing Cd levels and increasing patterns of free polyamines during nickel induced stress. Increasing polyamine patterns during Ni induced stress in poplar clone Pe19/66 are consistent with findings of increased foliar SPD and SPM levels in *Amaranthus paniculatus* plants during Ni induced oxidative stress [124], whereas significantly increased PUT levels were reported in *Brassica napus* under excess Ni accumulation [125]. Increased endogenous levels of PAs were also detected in the tissue culture of the commercial white poplar clone ‘Villafranca’ (*Populus alba*) after exposure to elevated Zn and Cu contents [49] and in poplar clones M1 (*Populus × euramericana*), PE19/66, and B229 (*Populus deltoides*) exposed to elevated soil Cu content [31]. Furthermore, elevated Zn amounts were found to increase the expression of the polyamine biosynthetic genes PaADC and PaODC in poplar leaves [49], while the addition of polyamines decreased the expression of genes encoding metallothionein type 2 (*PoMT2*) during Zn induced stress in *Plantago ovata* [126]. Tobacco (*Nicotiana tabacum*) leaves treated with CdCl_2_ showed increasing patterns of all free and conjugated polyamines, which contrasts with our finding that elevated Cd amounts reduce the main foliar and root polyamines in poplar [127]. When Mung bean was exposed to increased Cd content, putrescine levels increased, but spermidine and spermine levels decreased, which is consistent with our findings [128], whereas cadmium increased the enzyme activities of polyamine biosynthetic enzymes (ADC, ODC, SPMS, and SPDS) in *Oryza sativa* [129]. Mitigating effects of the exogenous application of SPD, SPM, and PUT during heavy metal induced stress were reported in wheat exposed to increased lead [130] and cadmium levels [131], which resulted in beneficial effects of polyamines, increased plant tolerance to heavy metals, and reduced metal phytotoxicity. 

Conjugated polyamines or phenylamides (PhA) are amides formed of aliphatic (e.g., putrescine, spermidine, or spermine) or aromatic (e.g., tyramine and tryptamine) polyamines and hydroxycimetic acids (most commonly caffeic, ferulic, and *p*-coumaric acid) [132]. As polyamines covalently linked to phenylpropanoids, phenylamides have the combined chemical properties of both components, providing them with a wide range of biochemical and metabolic actions, particularly related to free radical scavenging, so there are particularly involved in plant response to elevated heavy metal contents [46]. Because of the high levels of phenylpropanoids in poplars, these conjugated polyamines were abundant in poplar tissues [133]. In this study, all examined conjugated polyamines increased significantly at the root level when exposed to excess nickel, while conjugated polyamines prominently declined in both inspected organs after Cd treatment. These findings for cadmium response in poplar contrast to elevated amounts of conjugated polyamines found in *Hydrocharis dubia* when spermidine was applied exogenously to mitigate Cd induced stress [134]. Furthermore, when the same poplar clone was exposed to long-term effects of excess copper levels, increasing patterns of conjugated polyamines were observed, demonstrating the importance of conjugated polyamines in heavy metal tolerance in poplars [31].

## 4. Materials and Methods

### 4.1. Experimental Design and Sampling

In the experiment, 10 dm^3^ pots with sandy fluvisol soil were used (see Table 2). The substrate was artificially contaminated by separately adding Cd (NO_3_)_2_ and Ni (NO_3_)_2_ to final contents of 9 mg kg^−1^ Cd and 150 mg kg^−1^ Ni. The control substrate was not artificially contaminated. After the stabilization of the metal content via natural microbiological activity (which took eight weeks), non-rooted cuttings of *P. deltoides* clone PE 19/66 were planted in the spring in pots (including four cuttings in three replicates per treatment and the control) and grown in a greenhouse under semi-controlled conditions. The plants received regular irrigation and monthly additions of Hoagland’s solution. After five months of the experiment, one portion of the plant material (the leaves and roots) was used when it was fresh for the preparation of buffer extracts, while the second was frozen in liquid nitrogen and lyophilized to analyze plant hormones and the hormone regulator, and the third and fourth were dried at room temperature for radical scavenger capacity and TPC analyses and in the oven at 70 °C for the determination of metals content to achieve a constant weight, respectively.

### 4.2. Metal Content, Translocation (TF), and Bioconcentration Factors (BCF)

Using a microwave-assisted digestion system (model Milestone, D series), 300 mg of oven-dried and crushed plant material were digested with 10 mL of nitric acid and 2 mL of 30% *w/v* hydrogen peroxide and then diluted to 25 mL with deionized water. The samples were then processed using an Atomic Absorption Spectrophotometer (model FS AAS240/GTA120, Varian, California, CA, USA) and the acetylene/air burner flame technique with an atomization temperature of approximately 2300 °C. The contents of Mg, Ca, Cd, and Ni were determined at 285.2, 422.7, 228, and 232 nm, respectively, and expressed in mg kg^−1^ dry weight (DW) of plant material. All analyses of metal accumulation were performed in three biological and two technical replicates.

### 4.3. Activities of Antioxidant Enzymes, Radical Scavenger Capacity, Lipid Peroxidation Intensity and Content of Total Polyphenol Compounds 

To prepare buffer extracts of leaf and root samples for the measurement of antioxidant enzymes activities (POD, SOD, and GR), lipid peroxidation intensity (LP) and ferric reducing antioxidant power (FRAP test), 250 mg of fresh plant material was mixed with 2 mL of a 50 mM K-phosphate buffer (pH 7.0) using a ground glass homogenizer centrifuged at 15,000× *g*, and after separation of the supernatants, some were used for further analyses.

Seventy percent ethanol in a ratio of 1:10 (*w/v*) was added to 20 mg of air-dried plant material, and after centrifugation at 15,000× *g*, supernatants were used to test radical scavenger capacity (RSC) against DPPH, OH, NO radicals, and TPC.

All mentioned parameters were determined spectrophotometrically using a MultiScan spectrophotometer (Thermo Fisher Scientific, model Multiscan, Santa Clara, GO, CA).

(A) Enzymes activities (POD, SOD, and GR)

Guaiacol peroxidase (GPOD, EC 1.11.1.7) activity was measured according to Zimmerlin et al. [135] with minor modifications. Buffer extracts, as a source of POD, were added to the reaction medium with 0.1 M acetate buffer (pH 7.0) and 10 mM guaiacol as POD substrate. After the addition of 0.1 mM H_2_O_2_, the increase in the absorbance was measured at 436 nm over 2 min. The enzyme activity was calculated using the extinction coefficient for tetraguaiacol (e = 25.6 mM^−1^ cm^−1^) and expressed as enzyme units (U) g^−1^ FW, where one unit (U) represented the quantity of the enzyme that catalyzes the conversion of 1 μmol of substrate per min.

Superoxide dismutase (SOD) activity was determined by inhibiting the photochemical reduction of nitro blue tetrazolium (NBT) to formazan, which is a blue product of NBT reduction with superoxide anion (O2^•−^) [136]. Buffer extracts, as a source of SOD, were added to the reaction medium with 0.1 M K-phosphate buffer (pH 7.8), 13 mM methionine, 75 μM NBT, 0.1 mM EDTA, and 2 μM riboflavin. After the illumination of the samples using a fluorescent lamp for 10 min, a change in color was measured at 560 nm. The enzyme activity was defined as the amount of enzyme that inhibits NBT reduction by 50% at 25 °C and expressed as U g^−1^ FW.

Glutathione reductase (GR) activity was assayed using the Carlberg and Mannervik’s procedure [137]. Buffer extracts, as a source of GR, were added to the reaction medium with 100 mM phosphate buffer (pH 7), 1 mM GSSG, 1 mM EDTA, and 0.1 mM NADPH. Glutathione-dependent oxidization of NADPH was monitored for 2 min at 340 nm. The extinction coefficient was 6.22 mmol L^−1^ cm^−1^. The enzyme activity was expressed as U g^−1^ FW.

(B) Assays of Antioxidant Defense Systems

The DPPH-scavenging activity was determined according to Arnao et al.’s method [138] based on the reaction of the transformation of purple (λ_max_ = 515 nm) DPPH-radical (2,2-diphenyl-1-picrylhydrazyl) into reduced yellow form DPPH-H after incubation at 30 °C for 30 min in the dark. 

Neutralization of the hydroxyl radical (OH^•^) was determined by monitoring the degradation reaction of 2-deoxy-D-ribose in the presence of free OH^•^ radicals generated in the Fe^2+^/H_2_O_2_ system [139]. One of the final products of this reaction was malonyldialdehyde (MDA) which was determined spectrophotometrically with the help of a thiobarbiturate test (TBA test) at 532 nm. 

Nitric oxide (NO^•^) radical inhibition was calculated using the Griess Illosvory diazotization process and the method developed by Hensley et al. [140]. The chromophore’s absorbance was measured at 546 nm.

The total antioxidant power was measured using the FRAP assay [141] based on the reduction of Fe^3+^-TPTZ to Fe^2+^-TPTZ leading to a change in the reaction medium to a dark blue color with maximum absorbance at 593 nm.

Different concentrations of Trolox (6-Hydroxy-2,5,7,8-tetramethylchroman-2-carboxylic acid), which is a hydrophilic analog of vitamin E, were used as a standard in previously mentioned methods. Radical scavenger capacity against DPPH, OH, and NO radicals, as well as ferric reducing antioxidant power-FRAP were calculated using a standard curve and expressed as nmol of Trolox equivalents per g of fresh and dry weight of plant material (nmol TEAC g^−1^ FW/DW), depending on the extract used in the assay.

The intensity of lipid peroxidation (LP) was determined based on the content of malondialdehyde (MDA) as an end product of LP [142]. Absorbance was measured at 532 nm after incubation of the reaction medium (with the buffer extract and a solution containing 20% trichloroacetic acid and 0.5% 2-thiobarbituric acid) at 95 °C for 30 min. MDA amounts (determined by its molar extinction coefficient, 155 mM L^−1^ cm^−1^) were expressed as nmol MDA per gram fresh weight (nmol MDA g^−1^ FW).

The content of total phenolic (TPC) was estimated using the Folin–Ciocalteu assay according to a method developed by Singleton et al. [143] at 725 nm. The standard calibration curve was plotted using gallic acid and expressed as mg of gallic acid equivalents per g of dry weight of plant material (mg GAE g^−1^ DW).

### 4.4. Plant Hormones and Hormone Regulators Content

(A) Plant hormone analysis (ABA and IAA)

Freeze-dried leaves and roots weighing between 0.1 and 0.2 g DW were extracted using a solution of 65:35 isopropanol and 0.2 M imidazole buffer (pH 7.0). As an internal standard, [^13^C6]IAA and [^2^H4]ABA were added to the reaction mediums for the quantitative mass-spectral analysis of abscisic acid (ABA) and indole-3-acetic acid (IAA) in poplar leaves and roots. After overnight isotope equilibration, the analyses were performed according to Chen et al. [144] and Rapparini et al. [145] using gas chromatography-mass spectrometry-single ion monitoring (GC-MS-SIM) as described by Baraldi et al. [146]. The results of ABA and IAA quantification were expressed as ng g^−1^ DW.

(B) Polyamines determination

Plant tissues (approx. 20 mg DW of freeze-dried material) were extracted with 10 volumes of 4% perchloric acid (PCA). The homogenate was kept for 1 h on ice and then centrifuged at 15,000× *g* for 30 min. Aliquots of the supernatants and standard solutions of putrescine (PUT), spermidine (SPD), and spermine (SPM) were derivatized with dansylchloride as described by Scaramagli et al. [147]. Dansylated derivatives were extracted with toluene, dried, and resuspended in acetonitrile prior to HPLC analysis. Aliquots of the supernatant were subjected to acid hydrolysis (6 N HCl overnight at 110 °C) in order to release PAs from their PCA-soluble conjugates. Released PAs were derivatized and analyzed as described above. PAs were separated and quantified with HPLC (Jasco, Tokyo, Japan) using a reverse phase C_18_ column (Spherisorb ODS2, 5-μm particle diameter, 4.6 × 250 mm, Waters, Wexford, Ireland) and a programmed acetonitrile-water step gradient. The results of PAs quantification were expressed as nmol g^−1^ DW. The conjugated PAs amounts were calculated as the difference of total PAs in the hydrolyzed supernatants and the amounts of the free PAs in the nonhydrolyzed supernatants. 

### 4.5. Elemental Analysis of Nitrogen and Carbon Content

The contents of nitrogen (N) and carbon (C) in freeze-dried powdered poplar leaf material were determined using a CHN analyzer (model Elemental VARIO EL III, Hanau, Germany) coupled with a thermo-conductivity detector by using the manufacturer’s instructions. Acetanilide was used as a standard compound.

### 4.6. Statistics

Descriptive statistics, two factorial ANOVA, the *t*-test, principal component analysis (PCA), and Pearson correlation statistical techniques were employed. In two-way ANOVA, heavy metals (Ni and Cd) and plant organs (root and leaf) were used as factors, which were interpreted using the Fisher (F) test and their statistical significance levels. The *t*-test results were visually represented on a box plot diagram. The R programming environment was used for all statistical data processing (R Core Team). The “*rstatix*” R package [148] was used to calculate descriptive statistics and run two-way ANOVA and t-tests, while the “*ggplot2*” R package [149] was used for other visual representations. We used three levels of statistical significance throughout the paper denoted as (*) 0.05, (**) 0.01, (***) 0.001, and (****) 0.0001.

## 5. Conclusions

This study represents one of the first findings regarding the ability of the tested *P. deltoides* clone PE19/66 to be used for the phytoremediation of nickel and cadmium from soil. The study was designed to follow the effects of artificially applied heavy metals on poplar biological responses at the root and leaf levels. Tracking antioxidant, metabolic, and ROS enzymatic poplar biological responses and elevated nickel and cadmium soil amounts revealed a high metal- and -organ specificity. *P. deltoides* clone 19/66 showed an ambiguous response to different heavy metals, whereas polyamines showed mostly decreasing patterns of free polyamines in response to increased cadmium levels and increasing patterns of free polyamines in response to nickel induced stress. As a result of elevated nickel and cadmium soil contents, there was a significant increase in antioxidant activities, phenolic content, and ABA amounts at the poplar root level, confirming the role of this hormone in stress perception and signal transduction. These findings could be useful to develop afforestation programs for heavy metal-polluted habitats.

## Figures and Tables

**Table 1:** Metal and non-metal accumulation (mean ± SD), bioconcentration, and translocation factors of Ni and Cd in *P. deltoides* clone Pe19/66.

	Ni	Cd
Root metal accumulation (mg kg^−1^)	152.77 ± 8.01	17.46 ± 2.46
Leaf metal accumulation (mg kg^−1^)	23.36 ± 0.75	31.98 ± 2.52
Leaf calcium accumulation (mg kg^−1^)	11.86 ± 0.60	10.70 ± 0.29
Root calcium accumulation (mg kg^−1^)	8.24 ± 1.16	7.22 ± 0.97
Leaf magnesium accumulation (mg kg^−1^)	9.07 ± 0.29	8.35 ± 0.23
Root magnesium accumulation (mg kg^−1^)	5.55 ± 0.55	6.49 ± 0.99
Root bioconcentration factor (rBCF)	0.75	1.97
Aboveground bioconcentration factor (aBCF)	0.18	5.02
Translocation factor (TF)	24.62	261.8
Leaf nitrogen content (mg g^−1^)	19.9 ± 3.3	15.3 ± 1.4
Root nitrogen content (mg g^−1^)	9.75 ± 3.2	6.77 ± 1.2
Leaf carbon content (mg g^−1^)	420.5 ± 13.3	417.3 ± 12.5
Root carbon content (mg g^−1^)	391.3 ± 27.8	344.4 ± 10.8

**Table 2:** Chemical properties and particle size composition of the soil used in the experiment.

Horizon	Depth (cm)	pH (in H_2_O)	Humus (%)	CaCO_2_ (%)	Particle Size Composition					
					Coarse Sand (˃0.2)	Fine Sand (0.2–0.02)	Silt (0.02–0.002)	Clay (<0.002)	Total Sand (˃0.02)	Total Clay (<0.02)
Ap	0–30	7.55	2.64	17.08	0.5	37.4	40.4	21.7	37.9	62.1
I	30–58	7.91	1.58	19.56	0.3	45.9	34.8	19.0	46.2	53.8
II	58–72	8.08	1.00	16.06	0.3	71.0	15.9	12.8	71.3	28.7
III Geo	72–110	8.22	1.09	19.10	1.9	40.5	37.7	19.9	42.4	57.6
IV Geo	110–175	8.53	0.46	15.93	2.5	88.5	1.5	7.5	91.0	9.0

## References

[1] Yang W., Zhao F., Wang Y., Ding Z., Yang X., Zhu Z. (2020). Differences in Uptake and Accumulation of Copper and Zinc by Salix Clones under Flooded versus Non-Flooded Conditions. Chemosphere.

[2] Oyewo O.A., Adeniyi A., Bopape M.F., Onyango M.S., Prasad M.N.V., Pietrzykowski M. (2020). Chapter 4—Heavy Metal Mobility in Surface Water and Soil, Climate Change, and Soil Interactions. Climate Change and Soil Interactions.

[3] Zafar-ul-Hye M., Naeem M., Danish S., Fahad S., Datta R., Abbas M., Rahi A.A., Brtnicky M., Holátko J., Tarar Z.H. (2020). Alleviation of Cadmium Adverse Effects by Improving Nutrients Uptake in Bitter Gourd through Cadmium Tolerant Rhizobacteria. Environments.

[4] Shah K., Nongkynrih J.M. (2007). Metal Hyperaccumulation and Bioremediation. Biol. Plantarum..

[5] Ali H., Khan E., Ilahi I. (2019). Environmental Chemistry and Ecotoxicology of Hazardous Heavy Metals: Environmental Persistence, Toxicity, and Bioaccumulation. J. Chem..

[6] Singh D.J., Kalamdhad A. (2011). Effects of Heavy Metals on Soil, Plants, Human Health and Aquatic Life. Int. J. Res. Chem. Environ..

[7] Mahajan P., Kaushal J. (2018). Role of Phytoremediation in Reducing Cadmium Toxicity in Soil and Water. J. Toxicol..

[8] Genchi G., Carocci A., Lauria G., Sinicropi M.S., Catalano A. (2020). Nickel: Human Health and Environmental Toxicology. Int. J. Environ. Res. Public Health.

[9] Dixit P., Mukherjee P.K., Ramachandran V., Eapen S. (2011). Glutathione Transferase from Trichoderma Virens Enhances Cadmium Tolerance without Enhancing Its Accumulation in Transgenic Nicotiana Tabacum. PLoS ONE.

[10] Rao K.S., Mohapatra M., Anand S., Venkateswarlu P. (2010). Review on Cadmium Removal from Aqueous Solutions. Int. J. Eng. Sci. Technol..

[11] Chen M., Ogunseitan O.A., Wang J., Chen H., Wang B., Chen S. (2016). Evolution of Electronic Waste Toxicity: Trends in Innovation and Regulation. Environ. Int..

[12] Chen M., Qin X., Zeng G., Li J. (2016). Impacts of Human Activity Modes and Climate on Heavy Metal “Spread” in Groundwater Are Biased. Chemosphere.

[13] Kumar P., Fulekar M.H. (2019). Multivariate and Statistical Approaches for the Evaluation of Heavy Metals Pollution at E-Waste Dumping Sites. SN Appl. Sci..

[14] Moreira T.F.M., Santana I.L., Moura M.N., Ferreira S.A.D., Lelis M.F.F., Freitas M.B.J.G. (2017). Recycling of Negative Electrodes from Spent Ni-Cd Batteries as CdO with Nanoparticle Sizes and Its Application in Remediation of Azo Dye. Mater. Chem. Phys..

[15] Arya S., Kumar S. (2020). E-Waste in India at a Glance: Current Trends, Regulations, Challenges and Management Strategies. J. Clean. Prod..

[16] Nedjimi B. (2021). Phytoremediation: A Sustainable Environmental Technology for Heavy Metals Decontamination. SN Appl. Sci..

[17] Habibul N., Chen J.-J., Hu Y.-Y., Hu Y., Yin H., Sheng G.-P., Yu H.-Q. (2019). Uptake, Accumulation and Metabolization of 1-Butyl-3-Methylimidazolium Bromide by Ryegrass from Water: Prospects for Phytoremediation. Water Res..

[18] Kafle A., Timilsina A., Gautam A., Adhikari K., Bhattarai A., Aryal N. (2022). Phytoremediation: Mechanisms, Plant Selection and Enhancement by Natural and Synthetic Agents. Environ. Adv..

[19] Sun Y., Zhou Q., Wang L., Liu W. (2009). The Influence of Different Growth Stages and Dosage of EDTA on Cd Uptake and Accumulation in Cd-Hyperaccumulator (*Solanum Nigrum* L.). Bull. Environ. Contam. Toxicol..

[20] Baker A.J.M., McGrath S.P., Reeves R.D., Smith J.A.C., Terry N., Vangronsveld J., Banuelos G. (1999). Metal Hyperaccumulator Plants: A Review of the Ecology and Physiology of a Biological Resource for Phytoremediation of Metal-Polluted Soils. Phytoremediation of Contaminated Soil.

[21] Suman J., Uhlik O., Viktorova J., Macek T. (2018). Phytoextraction of Heavy Metals: A Promising Tool for Clean-Up of Polluted Environment?. Front. Plant Sci..

[22] Garbisu C., Alkorta I. (2001). Phytoextraction: A Cost-Effective Plant-Based Technology for the Removal of Metals from the Environment. Bioresour. Technol..

[23] Sarma H., Islam N.F., Prasad R., Prasad M.N.V., Ma L.Q., Rinklebe J. (2021). Enhancing Phytoremediation of Hazardous Metal(Loid)s Using Genome Engineering CRISPR–Cas9 Technology. J. Hazard. Mater..

[24] Matanzas N., Afif E., Díaz T.E., Gallego J.R. (2021). Phytoremediation Potential of Native Herbaceous Plant Species Growing on a Paradigmatic Brownfield Site. Water Air Soil Pollut..

[25] Yıldırım K., Kasım G.Ç. (2018). Phytoremediation Potential of Poplar and Willow Species in Small Scale Constructed Wetland for Boron Removal. Chemosphere.

[26] Malá J., Cvrčková H., Máchová P., Dostál J., Šíma P. (2010). Heavy Metal Accumulation by Willow Clones in Short-Time Hydroponics. J. For. Sci..

[27] Greger M., Landberg T. (1999). Use of Willow in Phytoextraction. Int. J. Phytoremed..

[28] Zacchini M., Pietrini F., Mugnozza G.S., Iori V., Pietrosanti L., Massacci A. (2008). Metal Tolerance, Accumulation and Translocation in Poplar and Willow Clones Treated with Cadmium in Hydroponics. Water Air Soil Pollut..

[29] Pajević S., Borišev M., Nikolić N., Krstić B., Pilipović A., Orlović S. (2009). Phytoremediation Capacity of Poplar (*Populus* spp.) and Willow (*Salix* spp.) Clonesin Relation to Photosynthesis. Arch. Biol. Sci..

[30] Wullschleger S.D., Weston D.J., DiFazio S.P., Tuskan G.A. (2013). Revisiting the Sequencing of the First Tree Genome: Populus Trichocarpa. Tree Physiol..

[31] Kebert M., Rapparini F., Neri L., Bertazza G., Orlović S., Biondi S. (2017). Copper-Induced Responses in Poplar Clones Are Associated with Genotype- and Organ-Specific Changes in Peroxidase Activity and Proline, Polyamine, ABA, and IAA Levels. J. Plant Growth Regul..

[32] Luo J.-S., Zhang Z. (2021). Mechanisms of Cadmium Phytoremediation and Detoxification in Plants. Crop. J..

[33] Ge W., Jiao Y.Q., Sun B.L., Qin R., Jiang W.S., Liu D.H. (2012). Cadmium-Mediated Oxidative Stress and Ultrastructural Changes in Root Cells of Poplar Cultivars. S. Afr. J. Bot..

[34] Li S., Yang D., Tian J., Wang S., Yan Y., He X., Du Z., Zhong F. (2022). Physiological and Transcriptional Response of Carbohydrate and Nitrogen Metabolism in Tomato Plant Leaves to Nickel Ion and Nitrogen Levels. Sci. Hortic..

[35] Chaoui A., El Ferjani E. (2005). Effects of Cadmium and Copper on Antioxidant Capacities, Lignification and Auxin Degradation in Leaves of Pea (*Pisum sativum* L.) Seedlings. Comptes Rendus Biol..

[36] Haider F.U., Liqun C., Coulter J.A., Cheema S.A., Wu J., Zhang R., Wenjun M., Farooq M. (2021). Cadmium Toxicity in Plants: Impacts and Remediation Strategies. Ecotoxicol. Environ. Saf..

[37] Shahzad B., Tanveer M., Rehman A., Cheema S.A., Fahad S., Rehman S., Sharma A. (2018). Nickel; Whether Toxic or Essential for Plants and Environment—A Review. Plant Physiol. Biochem..

[38] Seregin I.V., Kozhevnikova A.D., Davydova M.A., Bystrova E.I., Schat H., Ivanov V.B. (2007). Role of Root and Shoot Tissues of Excluders and Hyperaccumulators in Nickel Transport and Accumulation. Dokl. Biol. Sci..

[39] Cobbett C., Goldsbrough P. (2002). Phytochelatins and Metallothioneins: Roles in Heavy Metal Detoxification and Homeostasis. Annu. Rev. Plant Biol..

[40] Zimeri A.M., Dhankher O.P., McCaig B., Meagher R.B. (2005). The Plant MT1 Metallothioneins Are Stabilized by Binding Cadmiums and Are Required for Cadmium Tolerance and Accumulation. Plant Mol. Biol..

[41] Singh R., Gautam N., Mishra A., Gupta R. (2011). Heavy Metals and Living Systems: An Overview. Indian J. Pharmacol..

[42] Valko M., Morris H., Cronin M.T.D. (2005). Metals, Toxicity and Oxidative Stress. Curr. Med. Chem..

[43] Mittler R., Vanderauwera S., Gollery M., Van Breusegem F. (2004). Reactive Oxygen Gene Network of Plants. Trends Plant Sci..

[44] Danouche M., El Ghachtouli N., El Baouchi A., El Arroussi H. (2020). Heavy Metals Phycoremediation Using Tolerant Green Microalgae: Enzymatic and Non-Enzymatic Antioxidant Systems for the Management of Oxidative Stress. J. Environ. Chem. Eng..

[45] Santovito G., Trentin E., Gobbi I., Bisaccia P., Tallandini L., Irato P. (2021). Non-Enzymatic Antioxidant Responses of Mytilus Galloprovincialis: Insights into the Physiological Role against Metal-Induced Oxidative Stress. Comp. Biochem. Physiol. C Toxicol. Pharmacol..

[46] Velikova V.B., Edreva A.M., Tsonev T.D., Jones H.G. (2007). Singlet Oxygen Quenching by Phenylamides and Their Parent Compounds. Z. Naturforsch. C.

[47] Mandal C., Ghosh N., Maiti S., Das K., Gupta S., Dey N., Adak M.K. (2013). Antioxidative Responses of Salvinia (*Salvinia natans* Linn.) to Aluminium Stress and It’s Modulation by Polyamine. Physiol. Mol. Biol. Plants.

[48] Minocha R., Majumdar R., Minocha S.C. (2014). Polyamines and Abiotic Stress in Plants: A Complex Relationship. Front. Plant Sci..

[49] Franchin C., Fossati T., Pasquini E., Lingua G., Castiglione S., Torrigiani P., Biondi S. (2007). High Concentrations of Zinc and Copper Induce Differential Polyamine Responses in Micropropagated White Poplar (*Populus alba*). Physiol. Plant..

[50] Castiglione S., Todeschini V., Franchin C., Torrigiani P., Gastaldi D., Cicatelli A., Rinaudo C., Berta G., Biondi S., Lingua G. (2009). Clonal Differences in Survival Capacity, Copper and Zinc Accumulation, and Correlation with Leaf Polyamine Levels in Poplar: A Large-Scale Field Trial on Heavily Polluted Soil. Environ. Pollut..

[51] Song Y., Ci D., Tian M., Zhang D. (2014). Comparison of the Physiological Effects and Transcriptome Responses of Populus Simonii under Different Abiotic Stresses. Plant Mol. Biol..

[52] Gangwar S., Singh V.P. (2011). Indole Acetic Acid Differently Changes Growth and Nitrogen Metabolism in *Pisum sativum* L. Seedlings under Chromium (VI) Phytotoxicity: Implication of Oxidative Stress. Sci. Hortic..

[53] Elobeid M., Polle A., Gupta D., Sandalio L. (2012). Interference of Heavy Metal Toxicity with Auxin Physiology. Metal Toxicity in Plants: Perception, Signaling and Remediation.

[54] Asgher M., Khan M.I.R., Anjum N.A., Khan N.A. (2015). Minimising Toxicity of Cadmium in Plants-Role of Plant Growth Regulators. Protoplasma.

[55] Schwitzguébel J.-P., van der Lelie D., Baker A., Glass D., Vangronsveld J. (2002). Phytoremediation: European and American Trends. Successes, Obstacles and Needs. J. Soils Sediments.

[56] Sharma R.K., Agrawal M. (2005). Biological Effects of Heavy Metals: An Overview. J. Environ. Biol..

[57] Drążkiewicz M., Skórzyńska-Polit E., Krupa Z. (2003). Response of the Ascorbate–Glutathione Cycle to Excess Copper in *Arabidopsis thaliana* (L.). Plant Sci..

[58] Wang H., Shan X., Wen B., Zhang S., Wang Z. (2004). Responses of Antioxidative Enzymes to Accumulation of Copper in a Copper Hyperaccumulator of Commoelina Communis. Arch. Environ. Contam. Toxicol..

[59] He J., Ma C., Ma Y., Li H., Kang J., Liu T.-X., Polle A., Peng C., Luo Z.-B. (2012). Cadmium Tolerance in Six Poplar Species. Environ. Sci. Pollut. Res. Int..

[60] Hao F., Wang X., Chen J. (2006). Involvement of Plasma-Membrane NADPH Oxidase in Nickel-Induced Oxidative Stress in Roots of Wheat Seedlings. Plant Sci..

[61] del Carmen E.M., Souza V., Bucio L., Hernández E., Damián-Matsumura P., Zaga V., Gutiérrez-Ruiz M.C. (2002). Cadmium Induces Alpha(1)Collagen (I) and Metallothionein II Gene and Alters the Antioxidant System in Rat Hepatic Stellate Cells. Toxicology.

[62] Gajewska E., Skłodowska M. (2007). Effect of Nickel on ROS Content and Antioxidative Enzyme Activities in Wheat Leaves. Biometals.

[63] Gajewska E., Skłodowska M. (2005). Antioxidative Responses and Proline Level in Leaves and Roots of Pea Plants Subjected to Nickel Stress. Acta Physiol. Plant..

[64] Kısa D., Elmastaş M., Öztürk L., Kayır Ö. (2016). Responses of the Phenolic Compounds of Zea Mays under Heavy Metal Stress. Appl. Biol. Chem..

[65] Torres J., Barrientos E., Wrobel K., Wrobel K. (2014). Effect of Cadmium (Cd(II)), Selenium (Se(IV)) and Their Mixtures on Phenolic Compounds and Antioxidant Capacity in Lepidium Sativum. Acta Physiol. Plant..

[66] Schützendübel A., Schwanz P., Teichmann T., Gross K., Langenfeld-Heyser R., Godbold D.L., Polle A. (2001). Cadmium-Induced Changes in Antioxidative Systems, Hydrogen Peroxide Content, and Differentiation in Scots Pine Roots. Plant Physiol..

[67] Díaz J., Bernal A., Pomar F., Merino F. (2001). Induction of Shikimate Dehydrogenase and Peroxidase in Pepper (*Capsicum annuum* L.) Seedlings in Response to Copper Stress and Its Relation to Lignification. Plant Sci..

[68] Hale K.L., Tufan H.A., Pickering I.J., George G.N., Terry N., Pilon M., Pilon-Smits E.A.H. (2002). Anthocyanins Facilitate Tungsten Accumulation in Brassica. Physiol. Plant.

[69] Vuksanović V., Kovačević B., Stojnić S., Kebert M., Kesić L., Galović V., Orlović S. (2022). Variability of Tolerance of Wild Cherry Clones to PEG-Induced Osmotic Stress in Vitro. iForest.

[70] Król A., Amarowicz R., Weidner S. (2014). Changes in the Composition of Phenolic Compounds and Antioxidant Properties of Grapevine Roots and Leaves (*Vitis vinifera* L.) under Continuous of Long-Term Drought Stress. Acta Physiol. Plant..

[71] Solecka D., Kacperska A. (2003). Phenylpropanoid Deficiency Affects the Course of Plant Acclimation to Cold. Physiol. Plant..

[72] Kisa D., Kayir O., Saglam N., şahin S., Öztürk L., Elmastaş M. (2019). Changes of Phenolic Compounds in Tomato Associated With The Heavy Metal Stress. Bartın Univ. Int. J. Nat. Appl. Sci..

[73] Vuksanović V., Kovaćević B., Kebert M., Katanić M., Pavlović L., Kesić L., Orlović S. (2019). Clone Specificity of White Poplar (*Populus alba* L.) Acidity Tolerance in Vitro. Fresenius Environ. Bull..

[74] Puente-Garza C.A., Meza-Miranda C., Ochoa-Martínez D., García-Lara S. (2017). Effect of in Vitro Drought Stress on Phenolic Acids, Flavonols, Saponins, and Antioxidant Activity in Agave Salmiana. Plant Physiol. Biochem..

[75] Hamooh B.T., Sattar F.A., Wellman G., Mousa M.A.A. (2021). Metabolomic and Biochemical Analysis of Two Potato (*Solanum tuberosum* L.) Cultivars Exposed to In Vitro Osmotic and Salt Stresses. Plants.

[76] Gould K.S., Neill S.O., Vogelmann T.C. (2002). A Unified Explanation for Anthocyanins in Leaves?. Adv. Bot. Res..

[77] Kebert M., Trudić B., Stojnić S., Orlović S., Štajner D., Popović B., Galić Z. Estimation of Antioxidant Capacities of Poplar Clones Involved in Phytoremediation Processes. Proceedings of the STREPOW International Workshop.

[78] Kapoor D., Kaur S., Bhardwaj R. (2014). Physiological and Biochemical Changes in *Brassica juncea* Plants under Cd-Induced Stress. BioMed Res. Int..

[79] Siddiqui M.M., Abbasi B.H., Ahmad N., Ali M., Mahmood T. (2014). Toxic Effects of Heavy Metals (Cd, Cr and Pb) on Seed Germination and Growth and DPPH-Scavenging Activity in Brassica Rapa Var. Turnip. Toxicol. Ind. Health.

[80] Georgiadou E.C., Kowalska E., Patla K., Kulbat K., Smolińska B., Leszczyńska J., Fotopoulos V. (2018). Influence of Heavy Metals (Ni, Cu, and Zn) on Nitro-Oxidative Stress Responses, Proteome Regulation and Allergen Production in Basil (*Ocimum basilicum* L.) Plants. Front. Plant Sci..

[81] Metwally A., Safronova V.I., Belimov A.A., Dietz K.-J. (2005). Genotypic Variation of the Response to Cadmium Toxicity in *Pisum sativum* L.. J. Exp. Bot..

[82] Wu F.B., Zhang G.P., Dominy P. (2003). Four Barley Genotypes Respond Differently to Cadmium: Lipid Peroxidation and Activities of Antioxidant Capacity. Environ. Exp. Bot..

[83] Groppa M.D., Tomaro M.L., Benavides M.P. (2001). Polyamines as Protectors against Cadmium or Copper-Induced Oxidative Damage in Sunflower Leaf Discs. Plant Sci..

[84] Cho U.-H., Seo N.-H. (2005). Oxidative Stress in Arabidopsis Thaliana Exposed to Cadmium Is Due to Hydrogen Peroxide Accumulation. Plant Sci..

[85] Balestrasse K.B., Gallego S.M., Tomaro M.L. (2004). Cadmium-Induced Senescence in Nodules of Soybean (*Glycine max* L.) Plants. Plant Soil.

[86] Juknys R., Vitkauskaitė G., Račaitė M., Venclovienė J. (2012). The Impacts of Heavy Metals on Oxidative Stress and Growth of Spring Barley. Open Life Sci..

[87] Giannakoula A., Therios I., Chatzissavvidis C. (2021). Effect of Lead and Copper on Photosynthetic Apparatus in Citrus (*Citrus aurantium* L.) Plants. The Role of Antioxidants in Oxidative Damage as a Response to Heavy Metal Stress. Plants.

[88] Bartels D., Sunkar R. (2005). Drought and Salt Tolerance in Plants. Crit. Rev. Plant Sci..

[89] Tuteja N. (2007). Abscisic Acid and Abiotic Stress Signaling. Plant Signal. Behav..

[90] Danquah A., de Zelicourt A., Colcombet J., Hirt H. (2014). The Role of ABA and MAPK Signaling Pathways in Plant Abiotic Stress Responses. Biotechnol. Adv..

[91] Choudhary S.P., Bhardwaj R., Gupta B.D., Dutt P., Gupta R.K., Biondi S., Kanwar M. (2010). Epibrassinolide Induces Changes in Indole-3-Acetic Acid, Abscisic Acid and Polyamine Concentrations and Enhances Antioxidant Potential of Radish Seedlings under Copper Stress. Physiol. Plant..

[92] Yamasaki H., Cohen M.F. (2006). NO Signal at the Crossroads: Polyamine-Induced Nitric Oxide Synthesis in Plants?. Trends Plant Sci..

[93] Shevyakova N.I., Cheremisina A.I., Kuznetsov V.V. (2011). Phytoremediation Potential of Amaranthus Hybrids: Antagonism between Nickel and Iron and Chelating Role of Polyamines. Russ. J. Plant Physiol..

[94] Kishor P.K., Sangam S., Amrutha R.N., Laxmi P.S., Naidu K.R., Rao K.S., Rao S., Reddy K.J., Theriappan P., Sreenivasulu N. (2005). Regulation of Proline Biosynthesis, Degradation, Uptake and Transport in Higher Plants: Its Implications in Plant Growth and Abiotic Stress Tolerance. Curr. Sci. India.

[95] Siripornadulsil S., Traina S., Verma D.P.S., Sayre R.T. (2002). Molecular Mechanisms of Proline-Mediated Tolerance to Toxic Heavy Metals in Transgenic Microalgae. Plant Cell.

[96] Raghavendra A.S., Gonugunta V.K., Christmann A., Grill E. (2010). ABA Perception and Signalling. Trends Plant Sci..

[97] Bishnoi N.R., Sheoran I.S., Singh R. (1993). Influence of Cadmium and Nickel on Photosynthesis and Water Relations in Wheat Leaves of Different Insertion Level. Photosynthetica.

[98] Li S.-W., Leng Y., Feng L., Zeng X.-Y. (2014). Involvement of Abscisic Acid in Regulating Antioxidative Defense Systems and IAA-Oxidase Activity and Improving Adventitious Rooting in Mung Bean [*Vigna radiata* (L.) Wilczek] Seedlings under Cadmium Stress. Environ. Sci. Pollut. Res. Int..

[99] Haag-Kerwer A., Schäfer H.J., Heiss S., Walter C., Rausch T. (1999). Cadmium Exposure in Brassica Juncea Causes a Decline in Transpiration Rate and Leaf Expansion without Effect on Photosynthesis. J. Exp. Bot..

[100] Perfus-Barbeoch L., Leonhardt N., Vavasseur A., Forestier C. (2002). Heavy Metal Toxicity: Cadmium Permeates through Calcium Channels and Disturbs the Plant Water Status. Plant J..

[101] Sanità di Toppi L., Lambardi M., Pazzagli L., Cappugi G., Durante M., Gabbrielli R. (1998). Response to Cadmium in Carrot in Vitro Plants and Cell Suspension Cultures. Plant Sci..

[102] Chen S.L., Kao C.H. (1995). Glutathione Reduces the Inhibition of Rice Seedling Root Growth Caused by Cadmium. Plant Growth Regul..

[103] Hollenbach B., Schreiber L., Hartung W., Dietz K.J. (1997). Cadmium Leads to Stimulated Expression of the Lipid Transfer Protein Genes in Barley: Implications for the Involvement of Lipid Transfer Proteins in Wax Assembly. Planta.

[104] Sanità Di Toppi L., Lambardi M., Pecchion N., Pazzagli L., Durante M., Gabbrielli R. (1999). Effects of Cadmium Stress on Hairy Roots of Daucus Carota. J. Plant Physiol..

[105] Chen C.T., Chen L.-M., Lin C.C., Kao C.H. (2001). Regulation of Proline Accumulation in Detached Rice Leaves Exposed to Excess Copper. Plant Sci..

[106] Rubio M.I., Escrig I., Martinez-Cortina C., Lopez-Benet F.J., Sanz A. (1994). Cadmium and Nickel Accumulation in Rice Plants. Effects on Mineral Nutrition and Possible Interactions of Abscisic and Gibberellic Acids. Plant Growth Regul..

[107] Salt D.E., Rauser W.E. (1995). MgATP-Dependent Transport of Phytochelatins Across the Tonoplast of Oat Roots. Plant Physiol..

[108] Muñoz F.J., Dopico B., Labrador E. (1998). A CDNA Encoding a Proline-Rich Protein from Cicer Arietinum. Changes in Expression during Development and Abiotic Stresses. Physiol. Plant..

[109] Das P., Samantaray S., Rout G.R. (1997). Studies on Cadmium Toxicity in Plants: A Review. Environ. Pollut..

[110] Srivastava S., Srivastava A.K., Suprasanna P., D’Souza S.F. (2013). Identification and Profiling of Arsenic Stress-Induced MicroRNAs in *Brassica juncea*. J. Exp. Bot..

[111] Zelinová V., Alemayehu A., Bočová B., Huttová J., Tamás L. (2015). Cadmium-Induced Reactive Oxygen Species Generation, Changes in Morphogenic Responses and Activity of Some Enzymes in Barley Root Tip Are Regulated by Auxin. Biologia.

[112] Bhardwaj R., Sharma I., Handa N., Kapoor D., Kaur H., Gautam V., Kohli S., Anjum N.A., Gill S.S., Gill R. (2014). Role of polyamines in stress management. Plant Adaptation to Environmental Changes: Significance of Amino Acids and Their Derivatives.

[113] Saha J., Brauer E.K., Sengupta A., Popescu S.C., Gupta K., Gupta B. (2015). Polyamines as Redox Homeostasis Regulators during Salt Stress in Plants. Front. Environ. Sci..

[114] Benavides M.P., Groppa M.D., Recalde L., Verstraeten S.V. (2018). Effects of Polyamines on Cadmium- and Copper-Mediated Alterations in Wheat (*Triticum aestivum* L) and Sunflower (*Helianthus annuus* L) Seedling Membrane Fluidity. Arch. Biochem. Biophys..

[115] Shevyakova N.I., Il’ina E.N., Stetsenko L.A., Kuznetsov V.V. (2011). Nickel Accumulation in Rape Shoots (*Brassica napus* L.) Increased by Putrescine. Int. J. Phytoremediation.

[116] Pottosin I., Velarde-Buendía A.M., Bose J., Zepeda-Jazo I., Shabala S., Dobrovinskaya O. (2014). Cross-Talk between Reactive Oxygen Species and Polyamines in Regulation of Ion Transport across the Plasma Membrane: Implications for Plant Adaptive Responses. J. Exp. Bot..

[117] Velikova V., Yordanov I., Edreva A. (2000). Oxidative Stress and Some Antioxidant Systems in Acid Rain-Treated Bean Plants: Protective Role of Exogenous Polyamines. Plant Sci..

[118] Rahman H., Sabreen S., Alam S., Kawai S. (2005). Effects of Nickel on Growth and Composition of Metal Micronutrients in Barley Plants Grown in Nutrient Solution. J. Plant Nutr..

[119] Hasanuzzaman M., Nahar K., Fujita M., Anjum N.A., Gill S.S., Gill R. (2014). Regulatory role of polyamines in growth, development and abiotic stress tolerance in plants. Plant Adaptation to Environmental Changes: Significance of Amino Acids and Their Derivatives.

[120] Liu J.-H., Wang W., Wu H., Gong X., Moriguchi T. (2015). Polyamines Function in Stress Tolerance: From Synthesis to Regulation. Front. Plant Sci..

[121] Brüggemann L.I., Pottosin I.I., Schönknecht G. (1998). Cytoplasmic Polyamines Block the Fast-Activating Vacuolar Cation Channel. Plant J..

[122] Pál M., Horváth E., Janda T., Páldi E., Szalai G. (2006). Physiological Changes and Defense Mechanisms Induced by Cadmium Stress in Maize. J. Plant Nutr. Soil Sci..

[123] Chen L., Wang L., Chen F., Korpelainen H., Li C. (2013). The Effects of Exogenous Putrescine on Sex-Specific Responses of *Populus cathayana* to Copper Stress. Ecotoxicol. Environ. Saf..

[124] Pietrini F., Iori V., Cheremisina A., Shevyakova N.I., Radyukina N., Kuznetsov V.V., Zacchini M. (2015). Evaluation of Nickel Tolerance in *Amaranthus paniculatus* L. Plants by Measuring Photosynthesis, Oxidative Status, Antioxidative Response and Metal-Binding Molecule Content. Environ. Sci. Pollut. Res. Int..

[125] Shevyakova N.I., Il’ina E.N., Kuznetsov V.V. (2008). Polyamines Increase Plant Potential for Phytoremediation of Soils Polluted with Heavy Metals. Dokl. Biol. Sci..

[126] Raychaudhuri S.S., Pramanick P., Talukder P., Basak A., Atta-ur-Rahman (2021). Chapter 6-Polyamines, Metallothioneins, and Phytochelatins—Natural Defense of Plants to Mitigate Heavy Metals. Studies in Natural Products Chemistry.

[127] Kuthanová A., Gemperlová L., Zelenková S., Eder J., Machácková I., Opatrný Z., Cvikrová M. (2004). Cytological Changes and Alterations in Polyamine Contents Induced by Cadmium in Tobacco BY-2 Cells. Plant Physiol. Biochem..

[128] Choudhary A., Singh R.P. (2000). Cadmium-Induced Changes in Diamine Oxidase Activity and Polyamine Levels in Vigna Radiata Wilczek Seedlings. J. Plant Physiol..

[129] Pál M., Csávás G., Szalai G., Oláh T., Khalil R., Yordanova R., Gell G., Birinyi Z., Németh E., Janda T. (2017). Polyamines May Influence Phytochelatin Synthesis during Cd Stress in Rice. J. Hazard. Mater..

[130] Rady M., El-Yazal M., Taie H., Ahmed S. (2016). Response of Wheat Growth and Productivity to Exogenous Polyamines under Lead Stress. J. Crop. Sci. Biotechnol..

[131] Rady M.M., Hemida K.A. (2015). Modulation of Cadmium Toxicity and Enhancing Cadmium-Tolerance in Wheat Seedlings by Exogenous Application of Polyamines. Ecotoxicol. Environ. Saf..

[132] Bagni N., Tassoni A. (2001). Biosynthesis, Oxidation and Conjugation of Aliphatic Polyamines in Higher Plants. Amino Acids.

[133] Tsai C.-J., Harding S.A., Tschaplinski T.J., Lindroth R.L., Yuan Y. (2006). Genome-Wide Analysis of the Structural Genes Regulating Defense Phenylpropanoid Metabolism in Populus. New Phytol.

[134] Yang H.Y., Shi G.X., Li W.L., Wu W.L. (2013). Exogenous Spermidine Enhances Hydrocharis Dubia Cadmium Tolerance. Russ J. Plant Physiol..

[135] Zimmerlin A., Wojtaszek P., Bolwell G.P. (1994). Synthesis of Dehydrogenation Polymers of Ferulic Acid with High Specificity by a Purified Cell-Wall Peroxidase from French Bean (*Phaseolus vulgaris* L.). Biochem. J..

[136] Fridovich I. (1995). Superoxide Radical and Superoxide Dismutases. Annu. Rev. Biochem..

[137] Carlberg I., Mannervik B. (1985). Glutathione Reductase. Methods Enzymol..

[138] Arnao M.B. (2000). Some Methodological Problems in the Determination of Antioxidant Activity Using Chromogen Radicals: A Practical Case. Trends Food Sci. Technol..

[139] Halliwell B., Gutteridge J.M., Aruoma O.I. (1987). The Deoxyribose Method: A Simple “Test-Tube” Assay for Determination of Rate Constants for Reactions of Hydroxyl Radicals. Anal. Biochem..

[140] Hensley K., Mou S., Pye Q.N., Hensley K., Floyd R.A. (2009). Nitrite determination by colorimetric and fluorometric Griess diazotization assays. Methods in Pharmacology and Toxicology: Methods in Biological Oxidative Stress.

[141] Benzie I.F., Strain J.J. (1996). The Ferric Reducing Ability of Plasma (FRAP) as a Measure of “Antioxidant Power”: The FRAP Assay. Anal. Biochem..

[142] Devasagayam T., Boloor K., Ramasarma T. (2003). Methods for Estimating Lipid Peroxidation: An Analysis of Merits and Demerits. Indian J. Biochem. Biophys..

[143] Singleton V.L., Orthofer R., Lamuela-Raventós R. (1999). Analysis of Total Phenols and Other Oxidation Substrates and Antioxidants by Means of Folin-Ciocalteu Reagent. Methods Enzymol..

[144] Chen K.H., Miller A.N., Patterson G.W., Cohen J.D. (1988). A Rapid and Simple Procedure for Purification of Indole-3-Acetic Acid Prior to GC-SIM-MS Analysis. Plant Physiol..

[145] Rapparini F., Tam Y.Y., Cohen J.D., Slovin J.P. (2002). Indole-3-Acetic Acid Metabolism in Lemna Gibba Undergoes Dynamic Changes in Response to Growth Temperature. Plant Physiol..

[146] Baraldi R., Bertazza G., Bogino J., Luna V., Bottini R. (1995). Effect of Light Quality on Prunus Cerasus II. Changes in Hormone Levels in Plants Grown under Different Light Conditions. Photochem. Photobiol..

[147] Scaramagli S., Biondi S., Capitani F., Gerola P., Altamura M.M., Torrigiani P. (1999). Polyamine Conjugate Levels and Ethylene Biosynthesis: Inverse Relationship with Vegetative Bud Formation in Tobacco Thin Layers. Physiol. Plant..

[148] Kassambara A. (2021). Pipe-Friendly Framework for Basic Statistical Tests [R Package Rstatix Version 0.7.0].

[149] Wickham H. (2011). Ggplot2. WIREs Comput. Stat..

